# Chronic Obstructive Pulmonary Disease Increases the Risk of Mortality among Patients with Colorectal Cancer: A Nationwide Population-Based Retrospective Cohort Study

**DOI:** 10.3390/ijerph18168742

**Published:** 2021-08-19

**Authors:** Wei-Jen Cheng, Chih-Chao Chiang, Meng-Ting Peng, Yu-Tung Huang, Jhen-Ling Huang, Shang-Hung Chang, Hsuan-Tzu Yang, Wei-Chun Chen, Jong-Jen Kuo, Tsong-Long Hwang

**Affiliations:** 1 Center for Traditional Chinese Medicine, Chang Gung Memorial Hospital, Taoyuan 333, Taiwan; misterarren@gmail.com; 2 School of Traditional Chinese Medicine, College of Medicine, Chang Gung University, Taoyuan 333, Taiwan; 3 Graduate Institute of Clinical Medical Sciences, College of Medicine, Chang Gung University, Taoyuan 333, Taiwan; moonlight0604@hotmail.com; 4 Puxin Fengze Chinese Medicine Clinic, Taoyuan 326, Taiwan; 5 Oncology and Hematology Division, Department of Internal Medicine, Linkou Medical Center, Chang Gung Memorial Hospital, Taoyuan 333, Taiwan; p.mengting@gmail.com; 6 Center for Big Data Analytics and Statistics, Linkou Medical Center, Chang Gung Memorial Hospital, Taoyuan 333, Taiwan; anton.huang@gmail.com (Y.-T.H.); cratwoy0309@gmail.com (J.-L.H.); afen.chang@gmail.com (S.-H.C.); 7 Cardiovascular Division, Department of Internal Medicine, Linkou Medical Center, Chang Gung Memorial Hospital, Taoyuan 333, Taiwan; 8 College of Medicine, Chang Gung University, Taoyuan 333, Taiwan; lionsmanic@gmail.com; 9 Division of Thoracic and Cardiovascular Surgery, Department of Surgery, Linkou Medical Center, Chang Gung Memorial Hospital, Taoyuan, 333, Taiwan; b9205018@cgmh.org.tw; 10 Gynecologic Oncology Service, Department of Obstetrics and Gynecology, Linkou Medical Center, Chang Gung Memorial Hospital, Taoyuan 333, Taiwan; 11 Graduate Institute of Traditional Chinese Medicine, School of Traditional Chinese Medicine, College of Medicine, Chang Gung University, Taoyuan 333, Taiwan; 12 Research Center for Chinese Herbal Medicine, Research Center for Food and Cosmetic Safety, Graduate Institute of Health Industry Technology, College of Human Ecology, Chang Gung University of Science and Technology, Taoyuan 333, Taiwan; 13 Graduate Institute of Natural Products, College of Medicine, Chang Gung University, Taoyuan 333, Taiwan; 14 Department of Anesthesiology, Chang Gung Memorial Hospital, Taoyuan 333, Taiwan; 15 Department of Chemical Engineering, Ming-Chi University of Technology, New Taipei 243, Taiwan

**Keywords:** colorectal cancer, chronic obstructive pulmonary disease, overall survival, cancer registry, data linkage

## Abstract

*Background*: Colorectal cancer (CRC) is the third leading cause of cancer-related deaths in Taiwan. Chronic obstructive pulmonary disease (COPD) is associated with CRC mortality in several population-based studies. However, this effect of COPD on CRC shows no difference in some studies and remains unclear in Taiwan’s population. *Methods*: We conducted a retrospective cohort study using Taiwan’s nationwide database. Patients newly diagnosed with CRC were identified from 2007 to 2012 via the Taiwan Cancer Registry dataset and linked to the National Health Insurance research database to obtain their medical records. Propensity score matching (PSM) was applied at a ratio of 1:2 in COPD and non-COPD patients with CRC. The 5-year overall survival (OS) was analyzed using the Cox regression method. *Results*: This study included 43,249 patients with CRC, reduced to 13,707 patients after PSM. OS was lower in the COPD group than in the non-COPD group. The adjusted hazard ratio (aHR) for COPD was 1.26 (95% confidence interval (CI), 1.19–1.33). Moreover, patients with CRC plus preexisting COPD showed a higher mortality risk in all stage CRC subgroup analysis. *Conclusions*: In this 5-year retrospective cohort study, patients with CRC and preexisting COPD had a higher mortality risk than those without preexisting COPD, suggesting these patients need more attention during treatment and follow-up.

## 1. Introduction

Colorectal cancer (CRC) is a common and fatal malignancy, estimated to reach 2.5 million cases worldwide by 2035 [1]. In Taiwan, the incidence of CRC has rapidly increased during the past decades (2460 cases in 1988, 6679 cases in 1998, 11,449 cases in 2008, and 16,525 cases in 2018, as analyzed by the Taiwan Cancer Registry (TwCR)) [2]. The clinical presentations of CRC include no symptoms (the majority in the early stage), symptoms caused by the local tumor (bloody stool, abdominal pain, bowel habit change, or iron-deficiency anemia) [3], and symptoms from metastatic cancer (liver, lung, bone, brain, etc.) [4]. Patients with symptomatic CRC usually have advanced conditions and poor prognoses at diagnosis [5]. Asymptomatic patients with CRC diagnosed by screening have a better risk against death, recurrence, survival, and disease-free interval than those not diagnosed by screening [6]. Since 2008, the Ministry of Health and Welfare (MOHW) of Taiwan has started screening for CRC in the Cancer Care Quality Accreditation Program [2]. However, CRC remained the third leading cause of cancer death, leading to 6436 deaths (12.8% of all cancer deaths, 27.3 deaths per 100,000 population) in 2019, according to the MOHW of Taiwan [7].

Chronic obstructive pulmonary disease (COPD) is a chronic lung disease caused by increased resistance of the small conducting airways leading to emphysematous destruction in the lung. The chronic inflammatory pulmonary response to toxic particles and gases, such as tobacco usage, contributes to airway pathological change in patients with COPD [8,9]. The processes contributing to the small airway obstruction include disruption of the epithelial barrier, accumulation of inflammatory mucous exudates, infiltration of inflammatory cells, and deposition of connective tissue in the small airways [10]. This chronic inflammatory lung disease is found to be a risk factor for CRC incidence in Taiwan [11]. Similarly, in Korea, patients with COPD tend to have CRC, compared to those without COPD, irrespective of smoking habits [12]. As disease prognosis, CRC patients with preexisting COPD also require more medically intensive care, have a higher reoperation rate, and exhibit a worse 30-day mortality rate postoperatively than those without COPD [13]. Moreover, patients with a previous COPD diagnosis have more postoperative complications and adverse outcomes [14,15]. In a longer follow-up of elderly patients of European database with CRC, COPD is a significant comorbidity that causes death [16].

COPD patients and their comorbid conditions are in relation to overall clinical outcomes [17]. A review of epidemiological data revealed that patients with COPD have various comorbidities [18], and cardiovascular diseases are the most frequent comorbidity associated with COPD [19]. Some comorbidities are also associated with tobacco usage or caused by COPD, including coronary heart disease, congestive heart failure, and lung cancer. Other comorbidities, such as systemic venous thromboembolism, diabetes, metabolic syndrome, might be associated with extrapulmonary chronic systemic inflammation in COPD patients [20,21]. Apart from that, several COPD comorbidities, such as cardiovascular diseases and metabolic syndrome, are usually observed in senile people. As a hypothesis suggests, this results from COPD comorbidities sharing the same signaling pathways of aging [22].

However, the CRC genetic and exposure to risk factors may vary in individuals or populations from different ethnical origins. For example, a recent South African cohort study showed contrary results that comorbidities including COPD did not affect the survival of patients with CRC [23]. In the Taiwan population, there has been no research on mortality in patients with CRC plus COPD. Therefore, we conducted a retrospective observational cohort study using the Taiwan National Health Insurance Research Database (NHIRD) to uncover the association between COPD and CRC.

## 2. Materials and Methods

### 2.1. Data Source

This study’s data were obtained from the Health and Welfare Data Center (HWDC), including the NHIRD, TwCR data, and Causes of Death data, established by Taiwan’s MOHW. The HWDC, an official unit for research, is a data repository site that centralizes the NHIRD and about 70 other health-related databases for data management and analysis. All datasets under HWDC were encrypted, de-identified, and linkable.

The NHIRD was established by Taiwan’s National Health Insurance (NHI) Administration and has been used for various research and studies. Taiwan’s NHI program was conducted in 1995, which insured 99% of the 23.5 million Taiwanese people. The NHIRD contains encrypted healthcare utilization data from NHI beneficiaries, including information on NHI registry data and inpatient and ambulatory care claims, such as diagnoses of disease, surgical procedure, spending, and detailed prescriptions [24]. The TwCR is a population-based cancer registry system established by the MOHW in 1979 [25]. The TwCR has record completeness of 98.4%, and it has been regarded as one of the world’s highest-quality cancer registries [26]. Furthermore, since 2002, the TwCR has established a long-form database including cancer staging, detailed initial treatment, and recurrence information, which researchers have widely used for cancer recurrence studies [27]. In addition, the dataset provided demographic information and medical records of patients for further analysis, such as age, sex, date of birth, death, causes of death, cancer stage, recurrence condition, disease diagnosis, and inpatient and outpatient department prescriptions. The disease diagnosis for the current study was based on the *International Classification of Disease, Ninth Revision, Clinical Modification* (ICD-9-CM) from 2000 to 2015 or the ICD-10-CM from 2016, up to the end of the follow-up period.

### 2.2. Study Design and Population

This study used a retrospective cohort design. The latest data on the TwCR was from 2017, which was within the study duration. To investigate the 5-year overall survival (OS), we included patients newly diagnosed with CRC (ICD-9-CM code 153 and ICD-10-CM code C18) from 2007 to 2012. All medical records were reviewed since 2000 for clarification of COPD and other comorbidities. All patients from this cohort were followed up for five years. The exclusion criteria were invalid demographic data, missing cancer stage, and patients with CRC aged <20 years. COPD was identified as chronic bronchitis (ICD-9-CM code 491 and ICD-10-CM codes J41-J42), emphysema (ICD-9-CM code 492 and ICD-10-CM code J43), bronchiectasis (ICD-9-CM code 494 and ICD-10-CM code J47), and other types of chronic airway obstruction (ICD-9-CM code 496 and ICD-10-CM code J44). The diagnosis of COPD was made by pulmonologists with once hospitalization or twice visiting the outpatient department. Patients with CRC were classified into two groups according to the time of diagnosis of CRC and COPD. Any COPD diagnosed after CRC was excluded to avoid risk interference in this study. The study was conducted in accordance with the guidelines of the Declaration of Helsinki. The study protocol was reviewed by the Institutional Review Board of Chang Gung Memorial Hospital (IRB number 201900249B0) and approved on 4 March 2019. Any electronic information, patient identity, and organization were de-identified to protect patient privacy. Thus, the requirement for informed consent from patients was waived in this study.

### 2.3. Variables Definitions and Outcome Measurement

The major variables of our study cohort included age, sex, tumor-node-metastasis (TNM) cancer stage, and some clinical comorbidities. The pathological TNM stage from TwCR was classified into four categories based on the postoperative TNM classification. The clinical TNM stage was used if the pathological TNM stage was not available. In 2007–2009, the pathological TNM was recorded according to the sixth edition of the *Cancer Staging Manual* created by the American Joint Committee on Cancer (AJCC), whereas the seventh edition was used in 2010–2012. Disease comorbidities included diabetes mellitus (ICD-9-CM code 250 and ICD-10-CM codes E08-E13), hypertension (ICD-9-CM codes 401–405 and ICD-10-CM codes I10-I16), hyperlipidemia (ICD-9-CM code 272 and ICD-10-CM code E78), prior myocardial infarction (ICD-9-CM codes 410–412 and ICD-10-CM codes I21-I25), congestive heart failure (ICD-9-CM code 428, ICD-10-CM code I50), chronic kidney disease (ICD-9-CM codes 585–586, ICD-10-CM code N18), and liver disease (ICD-9-CM code 570–573, ICD-10-CM code K70-K77). For disease survival analysis, we linked TwCR to the cause of death data, which provided the underlying cause for death as diagnosed by physicians, and we included deaths related to cancer as the outcome events. All other causes of death, such as suicide, homicide, traffic accident caused, or non-cancer-related medical conditions were identified as non-outcome events (censoring).

### 2.4. Propensity Score Matching

We applied propensity score matching (PSM) to reduce the confounding effects of the two groups [28]. PSM with a ratio of 2:1 in the non-COPD and COPD groups with CRC was conducted based on the propensity scores of the probabilities accessed by age, sex, cancer stage, and comorbidities mentioned above. The matched group was used to examine the effects of COPD on the overall survival of patients with CRC.

### 2.5. Statistical Analysis

Descriptive data were analyzed before and after PSM to compare COPD and non-COPD patients with CRC stratified by demographic data and comorbidities. Pearson’s chi-square test and Student’s *t*-test were used for categorical and continuous variables, respectively. We used the Cox-adjusted survival curves to demonstrate the cumulative probability of OS in the two groups. Hazard ratios (HRs) with 95% confidence intervals (CIs) were calculated using the Cox proportional hazards regression model. Multivariate analysis of the Cox model was adjusted for age, sex, cancer stage, and comorbidities. A two-tailed *p*-value < 0.05 indicated a statistically significant difference. Data processing and analysis were conducted using SAS statistical software version 9.4 (SAS Institute Inc., Cary, NC, USA).

## 3. Results

### 3.1. Patient Characteristics

**Figure 1:**
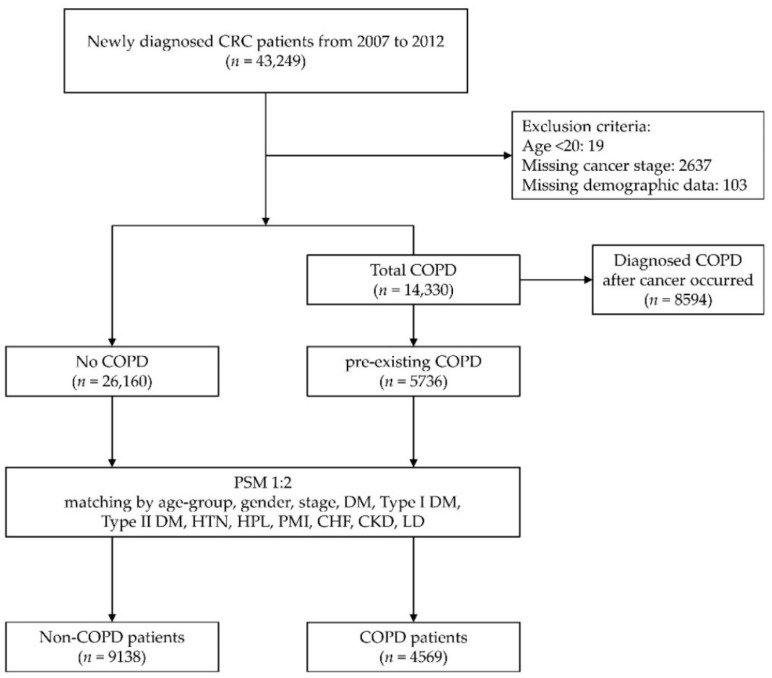
Flowchart of the patient enrollment process of this cohort study and variables used in propensity score matching. Abbreviations: CRC, colorectal cancer; COPD, chronic obstructive pulmonary disease; PSM, propensity score matching; DM, diabetes mellitus; HTN, hypertension; HPL, hyperlipidemia; PMI, prior myocardial infarction; CHF, congestive heart failure; CKD, chronic kidney disease; LD, liver disease.

Between 2007 and 2012, 43,249 patients newly diagnosed with CRC were included in this study. A total of 103 and 2637 patients were excluded because of missing demographic data or missing cancer stage records, respectively. Overall, 19 patients aged <20 years were excluded from this study. According to the COPD records, patients were classified into two groups—before or after CRC was diagnosed. A total of 14,330 (33.13%) of 43,249 patients with CRC suffered from COPD during the follow-up period. Upon excluding COPD after CRC diagnosis, we found that 5736 patients had underlying COPD before being diagnosed with CRC. After PSM at a ratio of 1:2, there were 4569 and 9138 COPD and non-COPD patients, respectively (Figure 1). Before matching, there was a total of 17,052 (53.46%) male and 14,844 (46.54%) female CRC patients. The average age was 65.3 years. The demographic data showed that patients with COPD were older (68.13% were >70 years), and most were male. Hypertension (total 17,659 patients; 55.36%) and diabetes mellitus (total 9168 patients; 28.74%) were the most common comorbidities in patients with CRC. We also found that patients with CRC plus COPD had more major comorbidities, such as prior myocardial infarction (44.61% versus 19.62%, *p* < 0.001), congestive heart failure (19.93% versus 6.02%, *p* < 0.001), chronic kidney disease (9.82% versus 4.79%, *p* < 0.001), and liver diseases (36.65% versus 26.8%, *p* < 0.001), compared with those without COPD. The pathological cancer stage was evenly distributed in stages 1 to 4 in both COPD and non-COPD groups. The characteristics before and after PSM are presented in Table 1.

### 3.2. Chronic Obstructive Pulmonary Disease Causes a Higher Mortality Rate in Patients with Colorectal Cancer

Since patients with COPD have more comorbidities and systemic disease, a European Eindhoven Cancer Registry study found that elderly patients with COPD have a worse OS [29]. Our data also revealed that patients with COPD were older and had more comorbidities than patients without COPD (Table 1). To reduce the effects of age and comorbidities between the study groups, we used PSM in a 2:1 ratio to reassign the study groups. After a 2:1 PSM, the variables of the investigated groups did not differ significantly, including gender, age group, cancer stage, and comorbidities (Table 1). Nevertheless, the overall age of the patients with COPD is higher than that of the patients without COPD (71.93 ± 11.33 versus 70.97 ± 10.72 years old), with a significant *p*-value but a non-significant absolute standard mean difference (ASMD = 0.09).

**Figure 2:**
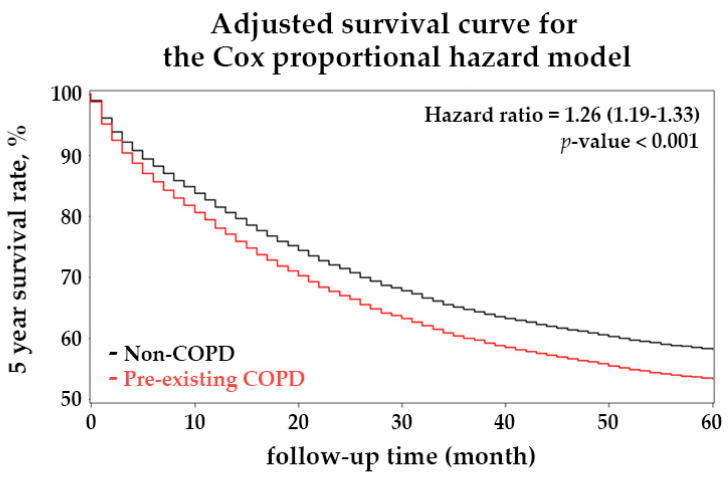
Five-year Cox-adjusted survival curves of patients with colorectal cancer (CRC) and with or without the preexisting chronic obstructive pulmonary disease (COPD) from this matched cohort study. Total 31,896 patients with CRC were matched by comorbidities using PSM with a 2:1 ratio, and the total number is reduced to 13,707 patients. Patients were followed up for 5 years after being diagnosed with CRC. The red line displays the Cox-adjusted survival curve of patients with CRC plus preexisting COPD, and the black line represents the control group.

**Figure 3:**
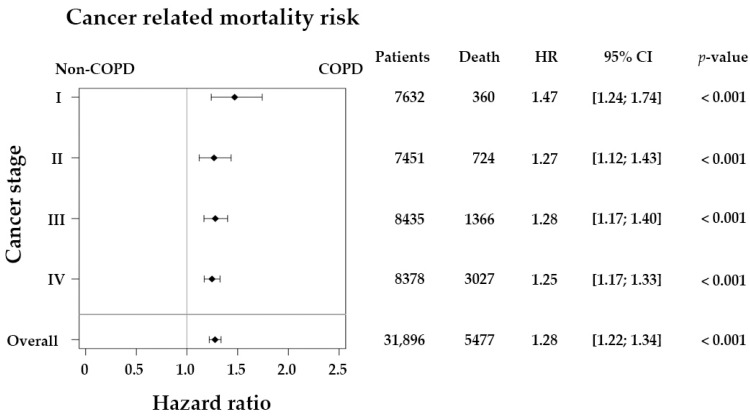
Subgroup analysis of cancer-related mortality with Cox regression model adjusted for sex and age-group by cancer stage, without propensity score matching. Total 31,896 patients with colorectal cancer were followed up for 5 years. The forest plot displays adjusted hazard ratios (HRs) and 95% confidence intervals (CIs) between preexisting chronic obstructive pulmonary disease (COPD) patients and non-COPD groups with different cancer stages by multivariate analysis.

Figure 2 illustrates the 5-year Cox-adjusted survival curves in the matched cohort. During the 5-year follow-up, patients with CRC and COPD had a significantly higher mortality risk than those in the non-COPD group (*p*-value < 0.001). Univariate and multivariate analysis for cancer-related mortality revealed an HR of 1.19 (95% CI 1.13–1.26) and 1.26 (95% CI 1.19–1.33), compared with the non-COPD group, respectively. Our work also indicates a lower mortality rate in young patients than in patients older than 70 (Table 2). Patients with stage IV CRC had a substantially higher mortality risk (HR, 17.25; 95% CI, 15.43–19.28) than patients with stage I CRC. Moreover, patients with comorbidities such as congestive heart failure (HR, 1.44; 95% CI, 1.30–1.59), chronic kidney disease (HR, 1.28; 95% CI, 1.15–1.42), and liver disease (HR, 1.08; 95% CI, 1.02–1.15) were associated with higher mortality risk. Notably, our work demonstrated that hyperlipidemia had a protective effect on cancer-related death in this cohort (HR, 0.82; 95% CI, 0.77–0.87). A PSM retrospective cohort study in Taiwan revealed a lower mortality risk for patients with gastric cancer among statin users [30]. For the subgroup analysis regarding the influence of COPD on OS in different CRC stages, the HR was 1.47 (95% CI 1.24–1.74) in stage I, 1.27 (95% CI 1.12–1.43) in stage II, 1.28 (95% CI 1.17–1.40) in stage III, and 1.25 (95% CI 1.17–1.33) in stage IV (Figure 3). These results showed that COPD increased the risk of patient mortality across all CRC stages.

## 4. Discussion

CRC accounts for approximately 10% of annually diagnosed malignancies and is the second and third most commonly diagnosed malignancy worldwide among females and males, respectively [1]. CRC caused approximately 881,000 deaths in 2018 and is the second leading cause of cancer-related deaths worldwide [31]. In Taiwan, CRC incidence has recently increased and is the third leading cause of cancer-related deaths [2,7]. Studies showed that chronic inflammatory lung disease, such as COPD, pneumoconiosis, and chronic rhinosinusitis, increased cancer incidence in Taiwan population-based cohort studies [11,32,33]. However, the relationship between COPD and CRC mortality has not yet been elucidated in Taiwan. In our study, patients with CRC and preexisting COPD had a higher mortality risk during the 5-year follow-up than those without preexisting COPD, according to our findings after PSM. Moreover, in all CRC stages, patients with preexisting COPD showed a higher mortality risk during the 5-year follow-up. Since COPD causes various health issues, we found that patients with CRC and preexisting COPD were older and had more comorbidities than those without preexisting COPD. Similarly, the elderly patients with CRC and COPD exhibited worse OS in a European Eindhoven Cancer Registry study [29]. Moreover, multiple comorbidities such as hypertension, diabetes mellitus, congestive heart failure, and chronic kidney disease are related to systemic inflammation, which is a poor prognostic factor for CRC [34,35], contributing to tumor invasion, cancer cell proliferation, and distant metastasis [36]. Approximately 20–40% of patients with CRC preoperatively have increased systemic inflammation markers, such as neutrophil-to-lymphocyte ratio and modified Glasgow prognostic score based on C-reactive protein [36]. As a result, older age and multiple comorbidities may contribute to worse mortality during the 5-year follow-up of patients with CRC and preexisting COPD.

The treatment of CRC would be decided by a multidisciplinary team including a pathologist, a gastrointestinal physician, a surgeon, an oncologist, and a radiologist, based on disease severity and patient condition [37]. Curative surgery is the most optimal treatment option for patients with resectable CRC [1]. However, COPD causes more surgical complications, such as cor pulmonale, respiratory distress, and pulmonary infections. In a Spanish population-based study, COPD was associated with higher rates of in-hospital complications, intensive care unit admission, antibiotic administration, reoperation, and mortality [15]. Furthermore, according to a Danish population-based study, postoperative death within 30 day increases among patients with CRC and COPD [13], which may affect the surgical decision. Nevertheless, the European Eindhoven Cancer Registry study did not show differences in surgery or chemotherapy choice in primary treatment with the adjusted Cox regression model despite the high risk of adverse outcomes in patients with COPD [29]. A limitation of our study is that the initial cancer treatment is not enrolled in the survival analysis. Thus, we do not know whether or not COPD would affect the initial cancer treatment decision in this cohort study or even lead to a higher mortality rate.

Interestingly, our 5-year cohort study revealed that patients with CRC plus hyperlipidemia had a lower mortality risk with multivariate analysis. Statins, or hydroxymethylglutaryl coenzyme A reductase inhibitors, are one of the most used drugs for treating hyperlipidemia in the world. This finding implied that statin might have a protective effect on patients with CRC. A long-term retrospective cohort study in Taiwan also demonstrated that statin usage could lower the mortality rate among patients with gastric cancer [30]. Furthermore, a meta-analysis in 2021 showed that statin treatment has protective effects against CRCs [38]. In CRC, statins were found to activate bone morphogenetic protein-specific phosphatase and tensin homolog, as well as inhibit the phosphoinositide 3-kinase/protein kinase B/mechanistic target of the rapamycin signaling pathway [39]. Therefore, statin treatment might explain why hyperlipidemia played a protective role in the cohort. However, the actual medicinal utilization for the treatment of hyperlipidemia was not accounted for in the analysis of our study. Further study may be needed to ascertain our hypothesis.

Our study had several strengths. First, the NHI program in Taiwan insured 99% of the 23.5 million Taiwanese people, and 93% of hospitals provided the NHI service, meaning that the TwCR and NHIRD data used in this study covered the entire population in Taiwan. Thus, there was no selection bias in enrolling subjects. Moreover, the number of study subjects was large, and the rate of cases lost to follow-up in this cohort was very low because the NHI program sustained a high coverage rate since its founding in 1995.

Our study also had some limitations. We initially excluded 2740 (6.34%) patients with CRC in this study due to missing cancer stage or demographic data. However, this retrospective study did not evaluate some confounding factors, including body mass index, smoking history, socioeconomic state, lifestyle, dietary habits, actual medication usage for comorbidities, location of CRC, tumor size, or initial cancer treatment, because these data were incomplete or unavailable. Thus, we could not know whether these patients have any treatment after initial diagnosis, which may influence the analysis results. Moreover, the severity of COPD was not evaluated in our study, which may be distinguished by admissions with acute exacerbation or medication usage, also affecting the mortality risk in patients with COPD. Finally, our data did not analyze the influence of initial cancer treatment, tumor location, and therapeutic modalities on overall survival, even though the cancer treatments are based on the cancer stage of CRC. Further detailed research is needed to elucidate these results.

## 5. Conclusions

In conclusion, patients with CRC and preexisting COPD had a higher mortality risk than those without preexisting COPD during this 5-year retrospective cohort study, suggesting these patients need more attention during treatment and follow-up. However, further detailed studies considering some confounding factors such as COPD severity or cancer therapeutic modalities are needed to confirm these findings.

## Figures and Tables

**Table 1:** Clinical characteristics before and after PSM of study patients.

Variables	Before Matching			After Matching ^a^		
	Non-COPD	COPD	*p*-Value *	Non-COPD	COPD	*p*-Value *
	(*n* = 26,160)	(*n* = 5736)		(*n* = 9138)	(*n* = 4569)	
**Sex**			<0.001			0.466
Female	12,506 (47.81)	2338 (40.76)		4102 (44.89)	2021 (44.23)	
Male	13,654 (52.19)	3398 (59.24)		5036 (55.11)	2548 (55.77)	
**Age, year (mean ± SD)**	63.47 ± 13.52	73.67 ± 11.04	<0.001	70.97 ± 10.72	71.93 ± 11.33	<0.001 ^b^
**Age-group, year**			<0.001			0.950
20–29	174 (0.66)	1 (0.02)		2 (0.02)	1 (0.02)	
30–39	1021 (3.9)	23 (0.40)		48 (0.53)	23 (0.51)	
40–49	2570 (9.82)	120 (2.09)		224 (2.45)	120 (2.63)	
50–59	6602 (25.22)	537 (9.36)		1040 (11.38)	537 (11.75)	
60–69	6703 (25.6)	1147 (20)		2299 (25.16)	1124 (24.6)	
>70	9090 (34.72)	3908 (68.13)		5525 (60.46)	2764 (60.49)	
**Cancer stage (pathology)**			<0.001			0.506
Stage I	6302 (24.09)	1330 (23.19)		2110 (23.09)	1093 (23.92)	
Stage II	6150 (23.51)	1301 (22.68)		2165 (23.69)	1037 (22.7)	
Stage III	6963 (26.62)	1472 (25.66)		2402 (26.29)	1194 (26.13)	
Stage IV	6745 (25.78)	1633 (28.47)		2461 (26.93)	1245 (27.25)	
**Comorbidity**						
DM	6900 (26.38)	2268 (39.54)	<0.001	3344 (36.59)	1671 (36.57)	0.980
Type I DM	266 (1.02)	97 (1.69)	<0.001	107 (1.17)	67 (1.47)	0.145
Type II DM	6702 (25.62)	2196 (38.28)	<0.001	3274 (35.83)	1619 (35.43)	0.650
Hypertension	13,233 (50.58)	4426 (77.16)	<0.001	6653 (72.81)	3286 (71.92)	0.273
Hyperlipidemia	8780 (33.56)	2648 (46.16)	<0.001	4066 (44.5)	2050 (44.87)	0.680
Prior myocardial infarction	5132 (19.62)	2559 (44.61)	<0.001	3026 (33.11)	1506 (32.96)	0.857
Congestive heart failure	1576 (6.02)	1143 (19.93)	<0.001	640 (7)	354 (7.75)	0.113
Chronic kidney disease	1252 (4.79)	563 (9.82)	<0.001	587 (6.42)	334 (7.31)	0.051
Liver diseases	7010 (26.8)	2102 (36.65)	<0.001	3002 (32.85)	1494 (32.7)	0.857

* Pearson’s chi-square test for categorical variables and Student’s *t*-test for continuous variables. ^a^ PSM was conducted with a 2:1 ratio on 31,896 patients with CRC by sex, age group, cancer stage, and comorbidities. ^b^ Absolute mean difference = 0.09, with no significant difference. Abbreviations: PSM, propensity score matching; SD, standard deviation; DM, diabetes mellitus; CRC, colorectal cancer; COPD, chronic obstructive pulmonary disease.

**Table 2:** Cox regression model with adjusted and non-adjusted hazard ratios and 95% confidence intervals of 5-year overall survival among patients with colorectal cancer in this matched cohort. (Total *n* = 13,707).

Variables	No. of Death	Univariate Analysis				Multivariate Analysis *			
		HR	95% CI		*p*-Value	HR	95% CI		*p*-Value
**COPD**									
No	3534	1 (reference)				1 (reference)			
Yes	1943	1.19	1.13	1.26	<0.001	1.26	1.19	1.33	<0.001
Sex									
Female	2469	1 (reference)				1 (reference)			
Male	3008	0.98	0.93	1.03	0.409	0.93	0.88	0.99	0.015
**Age-group, year**									
20–29	2	1.26	0.32	5.04	0.743	0.32	0.08	1.30	0.111
30–39	30	0.79	0.55	1.13	0.199	0.58	0.41	0.84	0.004
40–49	143	0.78	0.66	0.92	0.004	0.62	0.52	0.74	<0.001
50–59	449	0.48	0.44	0.53	<0.001	0.51	0.46	0.56	<0.001
60–69	1071	0.55	0.52	0.59	<0.001	0.60	0.56	0.64	<0.001
>70	3782	1 (reference)				1 (reference)			
**Cancer stage (pathology)**									
Stage I	360	1 (reference)				1 (reference)			
Stage II	724	2.21	1.95	2.51	<0.001	1.95	1.72	2.21	<0.001
Stage III	1366	4.02	3.58	4.52	<0.001	3.71	3.30	4.17	<0.001
Stage IV	3027	18.18	16.27	20.30	<0.001	17.25	15.43	19.28	<0.001
**Comorbidity**									
DM	2026	1.06	1.00	1.11	0.054	0.84	0.63	1.13	0.246
Type I DM	67	0.97	0.76	1.23	0.782	0.83	0.65	1.06	0.140
Type II DM	1979	1.06	1.00	1.12	0.039	1.33	1.00	1.78	0.053
Hypertension	3983	1.06	1.00	1.12	0.060	1.03	0.96	1.09	0.459
Hyperlipidemia	2214	0.78	0.74	0.82	<0.001	0.82	0.77	0.87	<0.001
Prior myocardial infarction	1743	0.95	0.89	1.00	0.057	0.97	0.91	1.03	0.283
Congestive heart failure	428	1.25	1.13	1.38	<0.001	1.44	1.30	1.59	<0.001
Chronic kidney disease	364	1.16	1.04	1.29	0.006	1.28	1.15	1.42	<0.001
Liver diseases	1712	0.90	0.85	0.95	<0.001	1.08	1.02	1.15	0.010

* Multivariate analysis was adjusted for COPD, age group, sex, cancer stage, DM, Type I DM, Type II DM, HTN, HPL, PMI, CHF, CKD, and LD. Abbreviations: HR, hazard ratio; CI, confidence interval; COPD, chronic obstructive pulmonary disease; DM, diabetes mellitus.

## Data Availability

The data presented in this study are available on request from the corresponding author. The data are not publicly available due to the data utilization policy of HWDC.
